# A Systematic Review of Neurological Complications in Pregnant Women With COVID-19

**DOI:** 10.7759/cureus.36388

**Published:** 2023-03-20

**Authors:** Mihirkumar P Parmar, Ritik Kathal, Sravani Bhavanam, Pranaya Baskaran, Nayanaa Varsaale, Bhavani Padamati, Hamsa Priya Bhuchakra, Mohammed Faseel C, Sweta Sahu, Shubha Davalgi

**Affiliations:** 1 Internal Medicine, GMERS Medical College and Hospital, Vadnagar, IND; 2 Internal Medicine, Shyam Shah Medical College, Rewa, IND; 3 Internal Medicine, Sri Devaraj Urs Medical College, Kolar, IND; 4 Internal Medicine, Government Theni Medical College, Theni, IND; 5 Internal Medicine, Capital Medical University, Beijing, CHN; 6 Internal Medicine, Atlantic University School of Medicine, Rodney Bay, LCA; 7 Internal Medicine, Apollo Institute of Medical Sciences and Research, Hyderabad, IND; 8 Internal Medicine, Government Medical College Kozhikode, Kozhikode, IND; 9 Surgery, JJM Medical College, Davanagere, IND; 10 Community Medicine, JJM Medical College, Davanagere, IND

**Keywords:** neurological manifestations, sars-cov-2, neurological complications, covid-19, pregnant women

## Abstract

Coronavirus disease 2019 (COVID-19), which is caused by the severe acute respiratory syndrome coronavirus 2 (SARS-CoV-2), has killed millions of people around the world so far and has turned into a disaster for people and healthcare systems. Neurological problems are often seen in people with COVID-19 in the general population, but it is unclear how common they are in pregnant women. This study provides a summary of studies on pregnant women with proven SARS-CoV-2 infection and a particular neurologic diagnosis from different parts of the world. After applying the inclusion and exclusion criteria, a total of 15 papers were assessed to create this review article. Based on our findings, the peripheral and central nervous systems were both equally impacted: Guillain-Barré syndrome (GBS, n=1), bifacial weakness, paresthesia, and vestibulocochlear neuritis (n=1), eclampsia types (n=2), and neurological disease (n=2); case reports, retrospective studies, editorials, and prospective observational studies were included. The median gestational age was 34 (30-36.5) weeks, and the median maternal age was 32.5 (25-35) years. Given the number of reports of neurologic problems associated with COVID-19 in the general community, our findings might be overstated, and we chose the ones that fit our criteria. We hope that this review helps in the early detection and management of neurological diseases during pregnancy.

## Introduction and background

Millions of people have been affected by the coronavirus disease 2019 (COVID-19) pandemic worldwide, including several pregnant women. As the pandemic continues and the research on it is ongoing, evidence has emerged to suggest that COVID-19 can result in neurological complications in some individuals. Though not much is known about the effects of COVID-19 on pregnancy, there are concerns that pregnant women may be at an increased risk of developing neurological complications from the disease. These neurological complications can have a significant impact on both the mother and the developing fetus.

In light of this, we conducted a systematic review of the neurological complications of COVID-19 in pregnant women to provide a comprehensive understanding of the potential neurological effects of COVID-19 on this subgroup. This study aims to synthesize existing research on the topic to identify the types and prevalence of neurological complications experienced by pregnant women with COVID-19, the potential risk factors associated with these complications, and the impact of these complications on maternal and fetal outcomes. The results of this study will provide critical information to healthcare professionals caring for pregnant women with COVID-19 and inform public health policies related to the management of COVID-19 in pregnancy.

According to the World Health Organization (WHO), pregnant women who are older, overweight, or who have a history of diseases like diabetes and hypertension are more at risk for having severe COVID-19 [1]. In general, 10% (7-12%) of screened pregnant and recently pregnant women have been diagnosed with COVID-19 [2]. However, population data analyses on pregnant women have found that the prevalence of COVID-19 among them was 2.2% in the USA and 1.03% in England [3,4].

Severe COVID-19 has been linked to pregnancy, especially in women with neurological problems like fatigue, myalgia, and headaches. Preeclampsia is a problem that can happen during pregnancy. It is linked to the severe acute respiratory syndrome coronavirus 2 (SARS-CoV-2) infection much more frequently, and the severity of COVID-19 is also linked to preeclampsia [5]. 

One of the most common signs of COVID-19 is a neurological symptom, such as tiredness or a headache. Anosmia and ageusia are the neurologic symptoms most specifically associated with COVID-19, but they are not very sensitive. Neurological problems are common and affect more than half of COVID-19 patients. They may also be a sign of other neurological problems. Even though it has not been seen very often in other coronaviruses, COVID-19 often affects both the central nervous system and the peripheral nervous system. COVID-19 has been linked to encephalopathy, Guillain-Barré syndrome (GBS), and stroke, which are some of the most common neurological problems [6]. Stroke and severe COVID-19 have been linked and delirium is a common encephalopathy symptom in pregnant women [7].

During pregnancy, the immune system's innate and adaptive responses change from pro-inflammatory to anti-inflammatory [8]. Also, physiological and immunomodulatory changes that happen during pregnancy may make COVID-19 symptoms worse, and pregnancy's physiological vulnerability to coronaviruses may make them more likely to get into the brain. Nevertheless, we were unable to locate any specific research on how COVID-19 in pregnancy might affect the nervous system [9]. 

This systematic review looks at the neurologic effects of COVID-19 reported during pregnancy and after delivery. It also provides a summary of the available information on the complexity of COVID-19 in pregnant women and women who have recently given birth. We have also looked into possible neurologic disease pathways that have been linked to COVID-19 in this subgroup of patients. 

Methods

**Figure 1:**
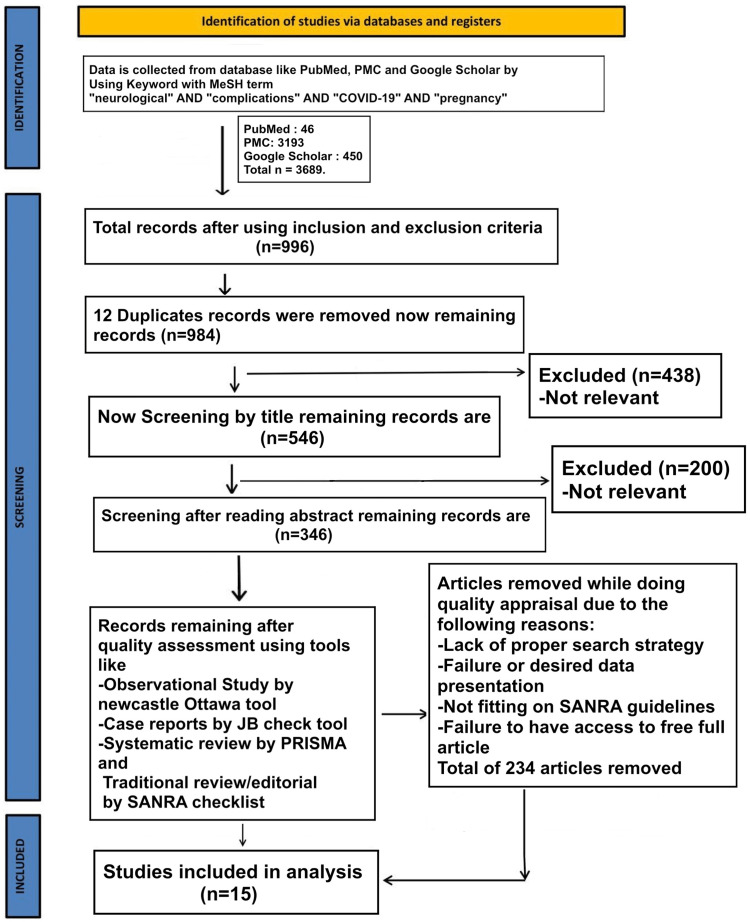
PRISMA flow chart PRISMA: Preferred Reporting Items for Systematic Review and Meta-Analysis; MeSH: Medical Subject Headings; SANRA: scale for quality assessment of narrative review articles; PMC: PubMed Central

We adhered to the Preferred Reporting Items for Systematic reviews and Meta-Analysis (PRISMA) guidelines for conducting our systematic review (Figure 1) [10].

We relied on the National Library of Medicine (PubMed), PubMed Central (PMC), and Google Scholar for collecting data through the use of the following medical subject headings (MeSH) terms with keywords such as "neurological" and "complications" and "COVID-19" and "pregnancy”. The total number of articles found on electronic databases was 3689.

Inclusion and Exclusion Criteria

In our analysis, we looked at all full-text papers, studies with people as subjects, and papers that were published in English. We located papers about COVID-19 neurological complications in pregnant women and pregnant COVID-19 female patients that were published in the last four years, from 2019 to 2022. Case reports, retrospective studies, editorials, and prospective observational studies were included.

We excluded research that did not involve people or articles without the full text. Systematic reviews, meta-analyses, and clinical reviews were not considered. Studies on neurological manifestations in men and non-pregnant female patients were also excluded.

Results

The papers were reviewed by each author, and those that were not relevant to our topic were manually removed. Only 15 papers were included in this systematic review. Table 1 provides a summary of 11 studies included in our analysis.

**Table 1:** A list of studies included in this review article COVID-19: coronavirus disease 2019; SARS-CoV-2: severe acute respiratory syndrome Coronavirus 2; SLE: systemic lupus erythematosus; CSF: cerebrospinal fluid; APLA: antiphospholipid antibodies

Citation	Year of publication	WHO Region	Country of study	Focus of the study	Findings	Key observations	Disease
Odeh et al. [5]	2022	Eastern Mediterranean Region	Palestine	Association of eclampsia in pregnant Women infected by COVID-19	The prevalence of eclampsia neurological symptoms is higher in pregnant women with COVID-19	Occipital headaches, nausea, visual changes	Atypical eclampsia
Mahajan et al. [6]	2021	Asia Region	India	COVID-19 has neurological side effects that impact both the central and peripheral nervous systems	The presence of autoimmune conditions in COVID-19 pregnant women could be triggering Guillain-Barré syndrome	An acute neurological complication of Guillain-Barré syndrome may be brought on by a pre-existing concomitant autoimmune disease like SLE or APLA	Guillain-Barré syndrome
Gama et al. [7]	2021	Region of the Americas	Brazil	Pregnant women with COVID-19 that progressed to neurological complications	COVID-19 affects the neurological function of the brain	A posterior round lesion in the cingulate gyrus that showed spontaneous hypersignal on T1 and spontaneous hypersignal with a hypointense halo on T2 was caused by a parenchymal hematoma	Stroke
Figueiredo et al. [8]	2020	European Region	Portugal	In pregnant women, peripheral facial paralysis is a COVID-19-presenting symptom	Acute facial nerve diseases that cause facial paralysis around the edges are often caused by viruses	COVID-19 was found to be the cause of an isolated peripheral facial palsy in a full-term pregnancy	Peripheral facial paralysis
Magalhães et al. [9]	2022	Region of the Americas	Brazil	Pregnancy and neurological complications of COVID-19	The central and peripheral nervous systems were equally affected in COVID-19 pregnant women	Given the predominance of ACE2, SARS-CoV-2 directly infects the brain by infecting the brain endothelium or nasal epithelial cells	Central and peripheral nervous system disease
Aasfara et al. [11]	2019	Eastern Mediterranean Region	Morocco	Bifacial paralysis, paresthesia, and vestibulocochlear neuritis in pregnant women as a result of COVID-19 manifestations	There are connections between vestibular neuritis, Guillain-Barré syndrome, and the SARS-CoV-2 infection	Nerve conduction investigations revealed an isolated absence of F waves in 2 nerves, together with albuminocytological separation and negative CSF SARS-CoV-2 RNA results (peroneal and tibial)	Bifacial weakness, paresthesia, and vestibulocochlear neuritis
Rodriguez et al. [12]	2020	European Region	Spain	A COVID-19 infection during pregnancy is a risk factor for neurological manifestations	SARS-CoV-2 infections could promote brain endothelial damage and facilitate neurological complications during pregnancy	Symptoms of cortical blindness and tonic-clonic seizures	Eclampsia
Syeda et al. [13]	2020	Regions of the Americas	USA	The clinical course of COVID-19 in pregnancy	COVID-19 causes CNS and PNS manifestations	Dizziness, headache, impaired consciousness, ataxia, and seizures	Neurological disease
Haghdoost and Goi [14]	2020	Eastern Mediterranean Region	Iran	Attention to neurological problems caused by COVID-19 in pregnancy	COVID-19 causes neurological problems in pregnant women	36% of people with COVID-19 have neurological symptoms like headaches, feeling lost, tingling in the hands and feet, and anxiety	Neurological disease
Sasaki et al. [15]	2022	Regions of the Americas	Brazil	CSF analysis of pregnant women at an early stage of COVID-19	Determine the actual entrance of SARS-CoV-2 into the central nervous system	Generalized tonic-clonic seizures, encephalopathy, anosmia, dysgeusia, headaches	Early neurological problems
Singh [16]	2020	Asia Region	India	COVID-19 in pregnancy with neurological symptoms vs. pregnancy with eclampsia	Pregnant women with COVID-19 and neurological manifestations mimicking eclampsia	36.4% of patients have headaches, seizures, or loss of consciousness	Neurological vs. eclampsia

## Review

For this scoping study, studies involving pregnant women with a confirmed SARS-CoV-2 infection who also had a specific neurologic disease from different parts of the world were gathered. We gathered 11 case records of COVID-19 neurologic problems in pregnant or postpartum women. It would be reasonable to assume that pregnant women in their second to fifth decades of life would not frequently be affected by neurologic complications of COVID-19 given that the majority of pregnant women are asymptomatic or only exhibit mild respiratory symptoms, as well as the fact that they are younger and have fewer comorbid conditions than nonpregnant women. Despite the fact that pregnant women are more likely than non-pregnant women to need ICU care and artificial breathing, the prognosis and mortality of COVID-19 appear to be similar in these patients. While our study found a connection between CNS involvement and ICU hospitalization, we could not find any studies regarding neurologic issues and pregnancy outcomes in COVID-19 patients.

Niraj et al. conducted a study involving 1572 COVID-19 pregnant women who were admitted to the hospital. It demonstrates that individuals with atypical preeclampsia incidences are those who manifest them before 20 weeks of gestation and after 48 hours following delivery, these are resistant to MgSO4 treatment and they tend to have HELLP syndrome. SARS-CoV-2 uses angiotensin-converting enzymes (ACE) as a receptor for entry into cells via a protein that binds to the N-terminal peptidase domain of ACE2 (which, according to recent studies, is expressed in placental cells). This enzyme plays a critical role in the renin-angiotensin-system (RAS), which controls blood pressure, angiogenesis, and embryonic development by controlling inflammation. Research has demonstrated that the virus spreads to cells that express the ACE2 receptor, including macrophages, fetal trophoblasts, and placental stromal cells. These receptors will be diminished as a result, and the RAS pathway will change. SARS-CoV-2, which modifies placental growth factor (PlGF) and excess placental soluble fms-like tyrosine kinase 1 (sFlt1) in infected placentas, may be the cause of preeclampsia. The virus causes multi-organ failure by inducing endothelial dysfunction, which is caused by an increase in antiangiogenic factor expression and a reduction in angiogenic factor expression. Another explanation for preeclampsia caused by SARS-CoV-2 is coagulopathy and fibrin buildup in the placenta of infected women. Preeclampsia incidence increased after previous research on other coronaviruses, such as Middle East Respiratory Disease (MERS) and SARS, was conducted. Common neurological symptoms of this virus range from headache, dizziness, and loss of taste and smell to severe cerebrovascular problems, encephalopathy, ataxia, skeletal muscle manifestations, viral encephalitis, seizures, and even death [5].

Even though there are many different kinds of GBS, the main symptoms are symmetrical limb weakness that gets worse quickly, areflexia when the patient is examined, sensory complaints, and in some cases, weakness in the face. GBS is also linked to a number of viral illnesses, such as influenza, Zika, SARS-CoV, MERS-CoV, and SARS-CoV-2. GBS is rare in pregnant women, and there is not much evidence to suggest that it can happen during pregnancy. In this case, autoimmune diseases like systemic lupus erythematosus (SLE) and antiphospholipid antibodies (APLA) may be the cause of GBS, which is a sudden neurological problem. One hypothesis for the pathogenesis is immune cross-reactions between host antigens and epitopes. Although the interval between a COVID-19 diagnosis and the beginning of GBS symptoms varies from case to case, it was a day shorter in this instance than it had been in other accounts in the literature. Consequently, GBS might be triggered by autoimmune illnesses during pregnancy and reveal itself early, as was the case in our case. As a result, it is advised that cases of COVID-19 with known or suspected autoimmune disease be especially evaluated for autoimmune neurological disease [6].

A study conducted in the US found that COVID-19 is a separate risk factor for ischemic cerebral vascular accidents (CVA). This shows that viral infection is linked to an increase in morbidity and mortality that goes beyond the disease's basic effects on the heart and lungs. Due to the possibility that SARS-CoV-2 could cause ACE2 to be turned down, COVID-19 has been linked to coagulopathies in very sick people, which can lead to bleeding. This can cause a narrowing of the blood vessels, a drop in the brain's ability to regulate itself, and pressure peaks, which can lead to bleeding and a rupture in the arterial wall. With an estimated frequency of 16.6 per 100,000 births, there is a higher chance of CVA happening during pregnancy as gestational age rises. Pregnancy-related CVA is the leading cause of significant long-term disability in postpartum women. It is estimated that 58.6% of cases are hemorrhagic, 28.4% are ischemic, 4.2% are CVTs, and 9.4% are strokes that are not otherwise diagnosed, with a death rate of 7.4% and a higher incidence in the puerperium. While little has been written on neurological issues in pregnant women infected with SARS-CoV-2, studies suggest that a subset may progress to multiple organ failure or death in addition to a higher risk of thrombotic events. Finally, it is yet unknown how typical CVA is among COVID-19 patients. Individuals with severe disease symptoms may have a higher chance of developing these vascular events. Although the clinical-radiological signs of autoimmune thrombophilia can vary, it is crucial to screen pregnant women for it [7].

A case report of pregnant women with COVID-19 admitted to the hospital by Gama et al. revealed that the SARS-structured CoV-2 resembles those of other recognized coronaviruses (CoV). SARS-CoV-2 can directly harm the nervous system since it has a high affinity for ACE2 receptors, which are found there. The highest virus titers shed from the nose are consistent with the highest expression of ACE2 receptors in the ciliated epithelium and goblet cells of the nasal mucosa, the olfactory nerve and bulb of SARS-CoV-2, which offers a direct route to the central nervous system. Peripheral facial palsy is more common in pregnant women, especially during the third trimester and the first week after giving birth. This susceptibility has been attributed to physiological changes during pregnancy, including a hypercoagulable state, increased cortisol levels, immunosuppression, an increase in total body water, changes in estrogen and progesterone levels, or conditions like hypertension, preeclampsia, and impaired glucose tolerance. Also, it appears that pregnant women have a worse functional prognosis for peripheral facial palsy, as their projected recovery is just 52% compared to 77%-88% in the non-pregnant, age-matched population. Corticosteroids are crucial for management since they reduce the likelihood of long-term impairments [8].

A hospital-based study found that COVID-19 leads to negative effects on both the central and peripheral nervous systems of pregnant women. The side effects of the infection, such as hypoxia, medication-related effects, toxins, metabolic abnormalities, or hyperimmune reactions brought on by the cytokine storm, may also produce COVID-19 neurologic problems. Since ACE2 is mostly expressed in both the nasal epithelial cells and the cerebral endothelium, it is possible that SARS-CoV-2 could infect the brain directly through either of these two places. Activation of glial cells and demyelination are thought to be caused by the pro-inflammatory state caused by the cytokine storm. So, immune responses may play a big role in the way the brain gets hurt, causing encephalopathies and encephalitis, especially in COVID-19 patients who get very sick. Also, when SARS-CoV-2 binds to ACE2, it stops the conversion of angiotensin II, a chemical that makes blood vessels narrow and causes inflammation. This is most likely how COVID-19 makes pregnant women more at risk for vascular issues, including preeclampsia-related vasomotor dysfunction [9].

Another case study involves a patient who had been infected with SARS-CoV-2 for six weeks and quickly got affected by facial paralysis on both sides, tingling in the extremities and inflammation of the right vestibulocochlear nerve. Clinical and electrophysiological findings were consistent with the bifacial weakness and paraesthesia (BFP) subtype of GBS. This study states that vestibulocochlear neuritis and BFP could also show up after infection with COVID-19. The acute immune-mediated neuropathy known as GBS has a variation called BFP. Axonal and/or demyelinating neuropathy is brought on by antibodies that cross-react with neuronal antigens as a result of molecular mimicry following an infection. Uncertainty exists regarding the underlying mechanisms of GBS in COVID-19. Although it can be a para-infectious occurrence or possibly a post-infectious immune-mediated process [11].

SARS-COV-2 infection during pregnancy may increase the risk of developing cancer-related posterior reversible leukoencephalopathy or preeclampsia/eclampsia syndrome because SARS-COV-2 has been found in brain endothelial cells, and eclampsia, posterior reversible leukoencephalopathy, and COVID-19 share a shared pathophysiology affecting endothelial tissue [12].

Syeda et al. conducted a study that examined 44,000 confirmed adult cases in hospitals. They found that headaches, dizziness, loss of consciousness, ataxia, and seizures are the most common signs of problems with the central nervous system. Peripheral nervous system manifestations include impairment of taste, vision, smell, and nerve pain. Skeletal muscular injury manifestations comprise skeletal muscle pain with an elevation in the serum creatinine kinase level. In a study of 214 non-obstetric patients with confirmed COVID-19 infection in China, 78 (36.4%) had neurologic manifestations [13].

Editorial work done by Haghdoost et al. suggests that the COVID-19 virus invades the immune system, causes persistent infection, and has neurological effects on the patient's nervous system and cognition. Due to the high risk of infection for pregnant women, COVID-19 could change the immune responses of both the mother and the fetus and cause serious harm to the health of both. During the COVID-19 outbreak, it is important to pay attention to the neurological and mental problems that pregnant women may have [14].

A prospective observational study among 14 pregnant women in a hospital by Sasaki et al. revealed that brain invasion by SARS-CoV-2 has been reported in human and animal models, but the route of entry of the virus to the blood-brain barrier (BBB) remains unclear. Since severe neurological manifestations of COVID-19 occur after the acute phase, microbiological examination of the CSF is rarely performed in the early stages of the disease. However, during the first wave of COVID-19, in CSF studies, samples of pregnant women whose symptoms had started between four and 18 days earlier were analyzed. The results indicate that SARS-CoV-2 is not present in the CSF of COVID-19 patients with early neurological symptoms, and therefore these symptoms may not be attributable to direct viral injury to the central nervous system [15].

A hospital-based retrospective study by Singh suggests that there are three possible scenarios: pregnancies with eclampsia without COVID-19; COVID-19 worsening preeclampsia or eclampsia; and pregnant women with COVID-19 and neurological symptoms mimicking eclampsia. The management differs in every case. In the first instance, treatment entails the management of cerebrovascular problems, investigation of CSF fluid, and clinical recommendations for COVID-19 in pregnancy. There are guidelines for managing eclampsia in the second case after excluding neurological problems brought on by COVID-19. Nonetheless, in both scenarios, proper infection control procedures and patient care should be provided in designated COVID-19 areas with the necessary safety precautions to safeguard the medical staff. In the third scenario, the patient's care must strictly adhere to the eclampsia management protocol [16].

Among COVID-19 individuals with neurologic symptoms, risk factors for CVD such as age, hypertension, and obesity are among the most prevalent comorbidities. None of the few case reports included in this comprehensive review employed CARE guidelines or other accepted standards for reporting scientific data. Thus, crucial information may be missing. Additionally, it has become clear that neurologic problems among pregnant women during the COVID-19 epidemic were underreported. As a result, a sizable fraction of young, healthy pregnant women may have developed modest neurological symptoms and complications from non-severe COVID-19. Additionally, the low detection rates of particular neurologic disorders may have been impacted by the overlap of preeclampsia and eclampsia symptoms.

## Conclusions

During our study, we came to the conclusion that the neurological symptoms that had been described before were most likely caused by SARS-CoV-2 infections. There is a possibility that the virus aggravated the damage that was already present in the endothelial cells of the brain. We believe that additional research is required to establish beyond a reasonable doubt that infection with SARS-CoV-2 during pregnancy raises a woman's chance of developing neurological issues during pregnancy. When everything is taken into consideration, it is essential to emphasize the significance of this point. It is critical to keep an eye out for any neurological symptoms associated with COVID-19 in pregnant women so that treatment can be started as soon as possible, thereby improving the patient's prognosis.

## References

[1] (2020). World Health Organization: COVID-19, pregnancy, and childbirth. https://www.who.int/news-room/q-a-detail/coronavirus-disease-covid-19-pregnancy-and-childbirth.

[2] Allotey J, Stallings E, Bonet M (2020). Clinical manifestations, risk factors, and maternal and perinatal outcomes of coronavirus disease 2019 in pregnancy: living systematic review and meta-analysis. BMJ.

[3] Gurol-Urganci I, Jardine JE, Carroll F (2021). Maternal and perinatal outcomes of pregnant women with SARS-CoV-2 infection at the time of birth in England: national cohort study. Am J Obstet Gynecol.

[4] Chinn J, Sedighim S, Kirby KA, Hohmann S, Hameed AB, Jolley J, Nguyen NT (2021). Characteristics and outcomes of women with COVID-19 giving birth at US academic centers during the COVID-19 pandemic. JAMA Netw Open.

[5] Odeh MA, Abuzneid YS, Badareen O, Masarweh K (2022). Atypical eclampsia in a pregnant woman infected by COVID-19. Case Rep Obstet Gynecol.

[6] Mahajan NN, Srivastava S, Chakor R, More P, Mahale SD, Gajbhiye RK (2021). Neurological complications of COVID-19 and spontaneous abortion in a pregnant woman - a case report. Eur J Obstet Gynecol Reprod Biol.

[7] Gama MD, Angelo Júnior JR, Cunha-Correia CD (2021). Stroke in COVID-19 and pregnancy: a case report. Rev Soc Bras Med Trop.

[8] Figueiredo R, Falcão V, Pinto MJ, Ramalho C (2020). Peripheral facial paralysis as presenting symptom of COVID-19 in a pregnant woman. BMJ Case Rep.

[9] Magalhães JE, Sampaio-Rocha-Filho PA (2022). Pregnancy and neurologic complications of COVID-19: a scoping review. Acta Neurol Scand.

[10] Page MJ, McKenzie JE, Bossuyt PM (2021). The PRISMA 2020 statement: an updated guideline for reporting systematic reviews. BMJ.

[11] Aasfara J, Hajjij A, Bensouda H, Ouhabi H, Benariba F (2021). A unique association of bifacial weakness, paresthesia and vestibulocochlear neuritis as post-COVID-19 manifestation in pregnant women: a case report. Pan Afr Med J.

[12] Garcia Rodriguez A, Marcos Contreras S, Fernandez Manovel SM, Marcos Vidal JM, Diez Buron F, Fernandez Fernandez C, Riveira Gonzalez MD (2020). SARS-COV-2 infection during pregnancy, a risk factor for eclampsia or neurological manifestations of COVID-19? Case report. BMC Pregnancy Childbirth.

[13] Syeda S, Baptiste C, Breslin N, Gyamfi-Bannerman C, Miller R (2020). The clinical course of COVID in pregnancy. Semin Perinatol.

[14] Haghdoost SM, Gol MK (2020). The necessity of paying more attention to the neurological and psychological problems caused by the COVID-19 pandemic during pregnancy. Int J Womens Health Reprod Sci.

[15] Sasaki LP, Fernandes GM, Silva AP (2022). Cerebrospinal fluid analysis of pregnant women at early stages of COVID-19. Taiwan J Obstet Gynecol.

[16] Singh S (2020). Coronavirus disease-2019 in pregnancy with neurological manifestations versus pregnancy with eclampsia: need for liberal testing to rule out the masquerades. Acta Obstet Gynecol Scand.

